# Comparison of PCR versus PCR-Free DNA Library Preparation for Characterising the Human Faecal Virome

**DOI:** 10.3390/v13102093

**Published:** 2021-10-18

**Authors:** Shen-Yuan Hsieh, Mohammad A. Tariq, Andrea Telatin, Rebecca Ansorge, Evelien M. Adriaenssens, George M. Savva, Catherine Booth, Tom Wileman, Lesley Hoyles, Simon R. Carding

**Affiliations:** 1 Gut Microbes and Health Research Programme, Quadram Institute Bioscience, Norwich Research Park, Norwich NR4 7UQ, UK; Shen-Yuan.Hsieh@quadram.ac.uk (S.-Y.H.); Andrea.Telatin@quadram.ac.uk (A.T.); Rebecca.Ansorge@quadram.ac.uk (R.A.); Evelien.Adriaenssens@quadram.ac.uk (E.M.A.); George.Savva@quadram.ac.uk (G.M.S.); Catherine.Booth@quadram.ac.uk (C.B.); Tom.Wileman@quadram.ac.uk (T.W.); Simon.Carding@quadram.ac.uk (S.R.C.); 2 Norwich Medical School, University of East Anglia, Norwich NR4 7TJ, UK; 3 Department of Biosciences, School of Science & Technology, Nottingham Trent University, Nottingham NG11 8NS, UK; lesley.hoyles@ntu.ac.uk

**Keywords:** virome, PCR bias, bacteriophage

## Abstract

The human intestinal microbiota is abundant in viruses, comprising mainly bacteriophages, occasionally outnumbering bacteria 10:1 and is termed the virome. Due to their high genetic diversity and the lack of suitable tools and reference databases, the virome remains poorly characterised and is often referred to as “viral dark matter”. However, the choice of sequencing platforms, read lengths and library preparation make study design challenging with respect to the virome. Here we have compared the use of PCR and PCR-free methods for sequence-library construction on the Illumina sequencing platform for characterising the human faecal virome. Viral DNA was extracted from faecal samples of three healthy donors and sequenced. Our analysis shows that most variation was reflecting the individually specific faecal virome. However, we observed differences between PCR and PCR-free library preparation that affected the recovery of low-abundance viral genomes. Using three faecal samples in this study, the PCR library preparation samples led to a loss of lower-abundance vOTUs evident in their PCR-free pairs (vOTUs 128, 6202 and 8364) and decreased the alpha-diversity indices (Chao1 *p*-value = 0.045 and Simpson *p*-value = 0.044). Thus, differences between PCR and PCR-free methods are important to consider when investigating “rare” members of the gut virome, with these biases likely negligible when investigating moderately and highly abundant viruses.

## 1. Introduction

The human intestinal microbiota is increasingly recognised as playing an essential role in health, contributing to digestion and the provision of essential micronutrients, and maintaining immune homeostasis and resisting infection [1,2]. It consists of complex dynamic communities of bacteria, viruses, fungi, archaea and protozoa among which viruses (the virome) dominate numerically [3,4,5,6].

The human intestinal virome comprises eukaryotic viruses, bacteriophages (phages) and viruses able to infect archaea in addition to endogenous retroviruses [4]. Phages (the phageome) make up more than 90% of the virome [3,5] with their ability to kill or alter the phenotype and function of their host cells enabling them to contribute to maintaining or disrupting intestinal homeostasis. The virome or phageome has been associated with infectious and autoimmune diseases such as HIV infection [7], childhood type 1 diabetes [8] and Crohn’s disease [9,10]. These and other disease associations have led to an increase in studies focusing on the intestinal virome although they are all constrained by the high genetic diversity and lack of universally conserved genes amongst viruses. Among culture-independent methods, metagenomics—a powerful sequence-based means of analysing the composition of the collective microbial genomes within an environmental sample—is currently the most reliable to survey the human virome.

Faecal samples are commonly used as a proxy for the intestinal virome based upon their accessibility and high biomass. One gram of faeces typically contains 10^8^ to 10^9^ virus-like particles (VLPs), the majority of which are tailed phages assigned to the class Caudoviricetes [11,12]. There is currently a lack of standardised protocols for virus isolation and sequencing, with few studies focusing on DNA and RNA viruses [13]. Size selection, precipitation and/or filtration methods are used to enrich samples for VLPs and to deplete extracellular macromolecules such as nucleic acids, proteins, carbohydrates, mureins, mucins and lipids. Virome-specific bioinformatic pipelines used to predict viral genomes, identify viral diversity, generate relative abundance and infer taxonomy are severely constrained by the paucity of reference viral genomes available in accessible databases [5,14,15].

A major challenge in studying the virome is low DNA yield either due to low viral biomass and/or inefficient extraction [16]. Low DNA yield can be overcome by DNA amplification using whole-genome amplification such as multi-displacement amplification (MDA) to generate the amounts required for generating sequencing libraries [17,18]. The disadvantage of amplification is that it can lead to amplification biases, such as for shorter inserts and over-amplification of small circular genomes [19,20]. The extent to which PCR-based amplification alone within sequencing library preparation contributes to sequencing bias has not been fully investigated. To address this important knowledge gap and identify potential confounders of virome characterisation we developed and used an optimised protocol for the isolation of VLPs from three human faecal samples. We then examined the PCR-associated bias by comparing a PCR-based and PCR-free workflow for library preparation prior to Illumina-based sequencing.

## 2. Materials and Methods

### 2.1. Faecal Sample Collection and Storage

This study was approved by the University of East Anglia (UEA) Faculty of Medicine and Health Sciences (FMH) Research Ethics Committee (FMH20142015-28), Norwich in 2014, and by the Health Research Authority (HRA) NRES Committee (17/LO/1102; IRAS: 218545) in 2017. Faecal samples were collected from three healthy adult males aged between 31 and 39 years of age with no use of antibiotics, probiotics or specific prebiotic products to enhance gut microorganisms in the 3-month period prior to sample collection. Samples were collected at the donors’ homes, followed by transportation at ambient temperature in an insulated container for delivery to the laboratory within 24 h of collection. Two of the samples were aliquoted after delivery and processed immediately (samples 1 and 2) and one after storage at −70 °C (sample 3).

### 2.2. Faecal VLP and VLP DNA Isolation

The methods for faecal VLP and VLP DNA isolation were adapted from published protocols [11,13,21]. Faecal aliquots (3–4 g) were homogenised in sterile TBT buffer (100 mM Tris-HCl, pH 8.0; 100 mM NaCl; 10 mM MgCl_2_·6H_2_O) [11] by vortexing, followed by chilling on ice for 1 h. The faecal homogenates were then centrifuged at 11,200× *g* for 30 min at 10 °C, followed by a second round of centrifugation under the same conditions. Supernatants were filtered sequentially through 0.8 µm (Sterlitech, Auburn, WA, USA) and 0.45 µm (Sartorius Ltd., Epsom, UK) PES cartridge filters. NaCl (final concentration 6%, *w*/*v*) was added to faecal filtrates, followed by adding PEG 8000 (final concentration 10%, *w*/*v*; Sigma-Aldrich Ltd., Gillingham, UK) to faecal filtrates. The samples were incubated at 4 °C for 16 h, followed by harvesting PEG-precipitated VLPs by centrifugation at 4500× *g* for 1 h at 4 °C and resuspending phage-containing pellets in ~500 μL of TBT buffer. VLP suspensions were treated with 10 U of TURBO DNase (Invitrogen/Thermo Fisher Scientific, Hemel Hempstead, UK) and 20 U of RNase I (Ambion/Thermo Fisher Scientific, Hemel Hempstead, UK) at 37 °C for 45 min, followed by addition of EDTA (final concentration 15 mM, pH 8.0) and heat inactivation at 75 °C for 10 min. Proteinase K (100 μg; Ambion/Thermo Fisher Scientific, Hemel Hempstead, UK) and 5% (*w*/*v*) SDS (final concentration 0.5%, *w*/*v*) were added to the samples and then incubated at 56 °C for 75 min, followed by addition of lysis buffer (final concentration 133.3 mM Tris-HCl, pH 8.0; 33.3 mM EDTA, pH 8.0; 3.3% SDS, *w*/*v*) and incubation at 65 °C for 15 min. An equal volume of phenol/chloroform/isoamyl alcohol (P/C/I, 25:24:1, *v/v/v*; Thermo Fisher Scientific, Hemel Hempstead, UK) was added to the VLP lysate and mixed thoroughly by vortexing for 30 s, followed by centrifugation at 15,000× *g* for 5 min at 20 °C, which was repeated once more. The resulting aqueous phase was then transferred to a ZR genomic DNA Clean & ConcentratorTM-25 column (Zymo Research; Cambridge Bioscience Ltd., Cambridge, UK) with the VLP DNA eluted in 50–70 μL of elusion buffer (low EDTA TE buffer, pH 8.0). To achieve high DNA concentrations, the aliquots of DNA from each of the same sample were pooled and concentrated (37–47 g in total; Table S1) using a SavantTM SpeedVac^®^ SC110 vacuum concentrator (Thermo Fisher Scientific, Hemel Hempstead, UK) with a SavantTM RT100 refrigerated condensation trap (Thermo Fisher Scientific, Hemel Hempstead, UK) to a final volume of 60–100 µL. Concentrated VLP DNA was stored at −70 °C until analysed. The quantity and quality of recovered VLP DNA were determined using the Nanodrop and QubitTM 1X dsDNA HS Assay Kit (Thermo Fisher Scientific, Hemel Hempstead, UK).

### 2.3. Bacterial Culture and Preparation of Phage Stock

*Bacteroides fragilis* (Bf) GB-124 [22], the reference host for the phage ΦB124-14 [23], was cultivated in Bacteroides Phage Recovery Medium (BPRM) [22] under anaerobic conditions (5% CO_2_, 5% H_2_ and 90% nitrogen at ~25 psi pressure) at 37 °C for 16–24 h. An aliquot (1 mL) of overnight culture was sub-cultured into fresh BPRM broth under anaerobic conditions at 37 °C and OD620 was checked every 30 min. Bacteria in mid-exponential phase (absorbance reached 0.3–0.33) were then used to propagate ΦB124-14 [23] for use in phage spiking recovery experiments as described previously [22]. In brief, an individual ΦB124-14 plaque was picked from a BPRM agar plate (1.5%, *w*/*v*) and then suspended in 200 μL of phage buffer (19.5 mM Na_2_HPO_4_, 22 mM KH_2_PO_4_, 85.5 mM NaCl, 1 mM MgSO_4_·7H_2_O, 0.1 mM CaCl_2_·2H_2_O; [23], followed by incubation at 4 °C for 16–24 h for phage propagation. The phage suspension was filtered using a 0.22 μm PES syringe filter (Sartorius Ltd., UK). This procedure was repeated up to three times using plaque assays to generate fresh plaques. Phage buffer (5–8 mL) was then added to the final agar plates and incubated at 20 °C for 1 h, followed by harvesting the liquid and top semi-solid agar layer (0.35%, *w*/*v*) into a 50-mL centrifuge tube (Corning, Ewloe, UK), mixing briefly and incubating at 20 °C for 30 min. The mixture was centrifuged at 3000× *g* for 20 min at 20 °C to remove bacterial debris and agar, followed by passing the supernatants through a 0.22 μm PES syringe filter and storing the filtrate at 4 °C. The titre of pure phage stocks was determined using plaque assays with serial dilutions from 10^−1^ to 10^−9^ prior to analysis (approximately 1 × 10^9^ to 1 × 10^10^ pfu/mL for use).

### 2.4. Plaque Assay-Based Phage Spiking and Recovery

To determine recovery of faecal VLPs using our optimised isolation protocol, faecal sample aliquots (3–4 g) were spiked with 1 mL of phage ΦB124-14 of known titre after homogenisation. Phage titres were subsequently determined after PEG precipitation for dual filtration (i.e., 0.8 µm combined 0.45 µm filter) using plaque assays. To perform plaque assays, 100 μL of 10-fold dilution series of spiked samples from 10^−1^ to 10^−9^ were mixed with 200 μL of the reference host GB-124 in 3 mL of semi-soft agar (0.35%, *w*/*v*) and poured on BPRM agar (1.5%, *w*/*v*). These agar plates were then incubated at 37 °C for 16–24 h in an anaerobic workstation, and pfu/mL determined.

### 2.5. Transmission Electron Microscopy (TEM)

Five microlitres of diluted faecal filtrate was added to a carbon-film on copper 400 mesh grid (EM Resolutions, Sheffield, UK) for 1 min and excess fluid removed by wicking the edge of the grid with Whatman filter paper, followed by incubation with 0.5% (*w*/*v*) uranyl acetate solution (BDH 10288) for 2 min. Excess liquid was removed using filter paper and the grid was dried. The grid was vapour-fixed by adding 1 mL of 2.5% (*v/v*) glutaraldehyde to a dish containing the dried grid for a minimum of 2 h. Faecal VLPs were viewed and captured using a Talos F200C TEM microscope at 200 kV with Gatan OneView digital camera.

### 2.6. Library Preparation and Shotgun Metagenomic Sequencing

Three independent VLP DNA samples were used for both PCR-based and PCR-free sequencing library preparation. In brief, input viral genomic DNA samples were randomly fragmented, followed by end-repairing, 5′-phosphorylation and A-tailing at 3′-end, and ligating adapters. Adapter-ligated DNA was then size-selected, followed using i5/i7 index primer sets to barcode, enrich and amplify the samples with PCR amplification for PCR-based libraries and with omitting PCR steps for non-amplified libraries. The six libraries were then sequenced using 2 × 150 bp paired-end chemistry (PE150) on the Illumina HiSeq X Ten platform (Novogene Ltd., Hong Kong). The raw data had a Q30 score of >90% for the libraries constructed generating 4.8–7.3 Gb of data per sample. Paired-end sequencing reads were provided as fastq format. All raw sequencing reads were pre-processed to trim and remove the reads having adapters, low quality (Q-value ≤ 38) and N nucleotides by Novogene Ltd., Hong Kong, using readfq [24] and Fxtools v0.17 [25]. Human genomic DNA identified by Kraken 2 (v2.0.8) [26,27] against the Genome Reference Consortium Human Build 37 database (GRCh37/hg19) was then removed using the confidence at 0.5 to reduce false positives, followed by further cleaning sequencing reads using fastp (v0.21.0) [28] with a quality cut-off of 20, prior to genome assembly. The extent of VLP enrichment was evaluated using ViromeQC (v1.0) in default mode [29].

### 2.7. Genome Assembly and Viral Genome Detection

Cleaned sequence reads were assembled using MEGAHIT assembler (v1.2.9) [30] with default parameters. QUAST (v5.0.2) [31] was used to assess the quality and quantity of assembled genomic contigs with default parameters. Potential viral contigs were then predicted using VirSorter (v1.0.3) [32] and VirFinder (v1.1) [33]. In this study, those putative viruses and proviruses sorted and classified into VirSorter categories 1 to 6 (including all predicted viruses and prophages) and those run through VirFinder under appropriate sorting conditions (score ≥ 0.7 and *p* < 0.05) were considered viral [5]. To access the completeness and qualities of sorted viral contigs, CheckV (v0.7.0) was used in end-to-end mode with default parameters [34].

### 2.8. Read Mapping, Cluster Analysis and Taxonomic Annotation

CD-HIT-EST (v4.8.1) [35,36] was used to produce a pooled, non-redundant viral contig file across all datasets using specific parameters (-p 1 -g 1 -aS 0.9 -c 0.95 -M 0 -T 0), resulting in viral operational taxonomic units (vOTUs) clustered at 95% sequence identity over 90% of the contig length. Each vOTU was represented in the dataset by the longest contig of a uncultivated virus genomes (UViGs). The viral reads were then mapped to the UViGs representing vOTUs using BWA (v0.7.17) with the bwa-mem mode for paired-end manner [37], followed by SAMtools (v1.10) to sort and index [38]. Cluster analysis to taxonomically classify the UViGs representing vOTUs was carried out using vConTACT 2.0 [39,40]. Prodigal (v2.6.3) [41] was used to detect open reading frames on UViGs. The ProkaryoticViralRefSeq94-merged database was used alongside using the diamond mode and ClusterONE mode flags. The output was visualised using Cytoscape [42]. In parallel, Demovir [43] was applied to assign taxonomy to the vOTUs using default parameters. For those viral genomes that could not be identified and annotated by Demovir, vConTACT 2.0 was then used to annotate the remaining unknown sequences at the family level by manual curation of the network clusters using taxonomic information from the Master Species List (MSL36) [44]. If neither tool was able to assign taxonomy, the viral genomes were labelled as “unassigned”.

### 2.9. Analysis of Relative Abundance, Alpha and Beta Diversity and Statistical Analysis

Analysis of relative abundances and alpha- and beta-diversity were performed on the non-redundant contig catalogue (vOTUs; clustered at 95% sequence identity) and metagenomic reads mapping to it (see above). We retrieved per-vOTU read coverage from the mean of per-base coverage which we rounded to the closest integer to obtain count-like data. For the following analyses we only considered vOTUs longer than 1 kbp. We created a PhyloSeq object from these data and rarefied the counts to the sample with the lowest count (102,756) using tidyverse (v1.3.0) [45] and PhyloSeq (v1.30.0) [46]. The observed species richness, Chao1, Shannon and Simpson alpha diversity indices were calculated using the plot-richness function on the rarefied data in PhyloSeq.

For the following analyses, all vOTUs that did not have more than three counts in at least one sample were removed and the non-rarefied counts transformed into relative abundances by dividing each count by sample sums. These data were subsampled to the top 25 vOTUs with highest sums of counts across samples and visualized as a bubble plot. Principal coordinate analysis (PCoA) plots were generated on Bray–Curtis dissimilarities of relative abundances using the plot_ordination function of PhyloSeq. An additional PCoA plot was obtained based on relative abundances with a more stringent filtering performed to remove low abundant vOTUs with relative abundances below 0.5% in all samples. For statistical analysis of the data paired *t*-tests were performed and data plots were created in R.

## 3. Results

### 3.1. VLP and DNA Extraction Protocol

**Figure 1:**
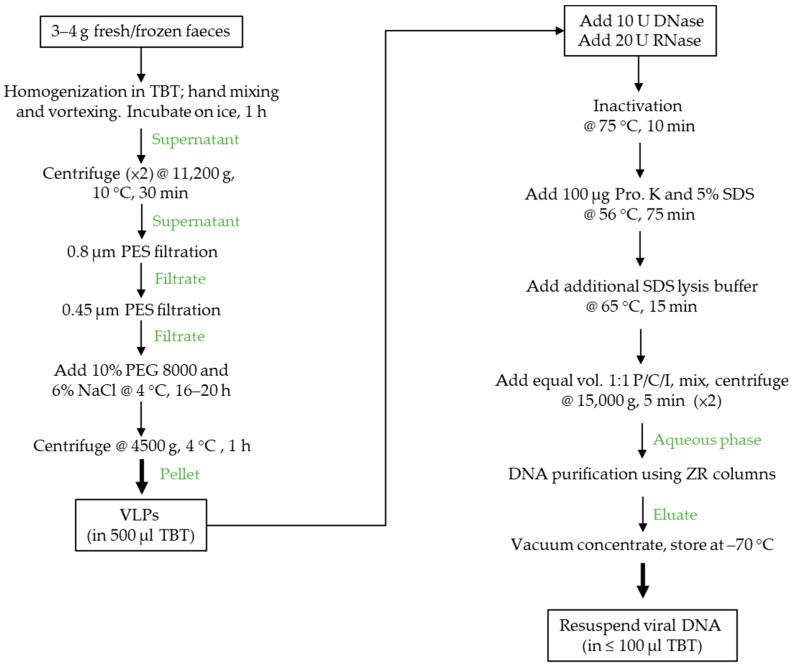
Workflow of the VLP isolation and VLP DNA extraction protocol. Three- to four-gram aliquots of faecal material were homogenised, centrifuged to remove debris and large particulate matter and then sequentially filtered through 0.8 µm and 0.45 µm cartridge PES filters to remove remaining contaminants and bacteria followed by enrichment of VLPs by PEG precipitation. Recovered VLPs were treated with DNase/RNase to remove non-capsid-associated nucleic acids prior to disrupting viral capsids with proteinase K/SDS. Released capsid nucleic acids were treated with P/C/I prior to column purification. DNA was eluted, vacuum concentrated to 60–100 µL and then stored at −70 °C prior to use.

Faecal samples (two fresh and one frozen) obtained from three healthy unrelated adult donors were used to develop an efficient VLP/DNA isolation protocol and a bioinformatics pipeline for VLP-enriched virome analysis to investigate PCR-associated biases in Illumina sequencing datasets (Figure 1). In developing the VLP isolation protocol, we evaluated and modified published protocols by combining key steps and adopting dual filtration [11,13,21]. To assess the efficiency of VLP recovery, samples were spiked with the reference phage ΦB124-14 to quantify phage recovery by plaque assays and epifluorescence microscopy (EFM; see Method S1 in Supplementary Materials). Overall, VLP recovery assessed by spiking samples with ΦB124-14 was 30–40% (35.8 ± 3.96%, mean ± SD, *n* = 3) as determined by plaque assays (Table S1) and ~96% by EFM. VLP DNA yields were between 67.2 ng/g and 94.8 ng/g of faeces. Moreover, all three DNA samples were of high quality with low levels of protein contamination based on A_260_:A_280_ ratios (Table S1) with gel electrophoresis showing that all samples contained a major DNA product above or close to the 48.5 kbp molecular weight marker with less intense staining of DNA smears indicative of low levels of DNA of other fragment sizes or shearing of the DNA.

### 3.2. Transmission Electron Microscopy (TEM)

TEM images after dual filtration showed the majority of faecal VLPs were bacteriophages (Figure S1), including siphoviruses with isometric heads ranging from 50 nm to 200 nm in diameter and various tail lengths of between 180 nm and 600 nm (S1 panels A−C). Different morphotypes of myovirus-like VLPs were also observed, with icosahedral heads of ~100 nm in diameter and diverse tail lengths, ranging from 100 to 200 nm (S1 panels D and E). Some myovirus virions were observed with their tails attached to membranous-like materials (S1 panels D and E). In addition, many separate viral capsids and tails were found in these samples, consistent with 0.45 µm filtration negatively affecting structural stability of viral particles. However, this step was effective in removing solid materials as well as bacterial cells.

### 3.3. Viromics Pipeline

**Figure 2:**
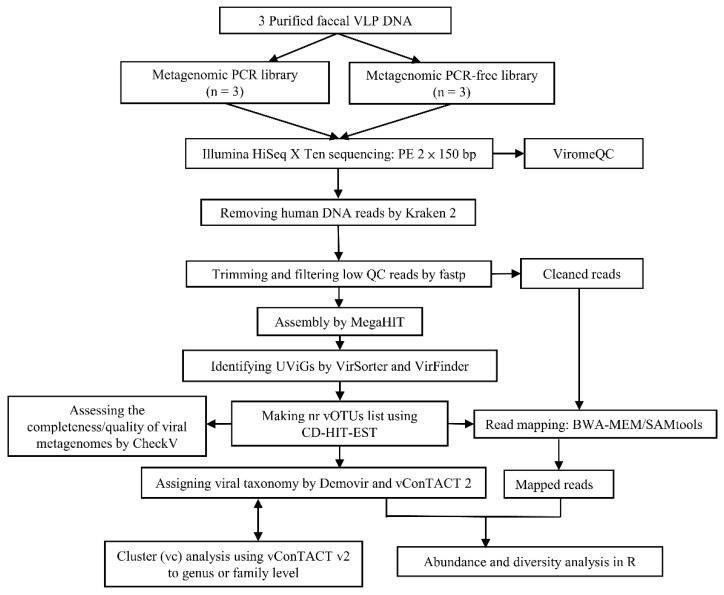
Overview of bioinformatic pipeline for cross-comparative virome study. Human DNA sequences identified by Kraken 2 against GRCh37 (Genome Reference Consortium; hg19) were removed, followed by trimming and filtering low quality reads using fastp prior to de novo assembly. MEGAHIT was used to assemble Illumina short reads into longer contigs, followed by detecting potential uncultivated viral genomes (UViGs) candidates using VirSorter and VirFinder. UViGs were used to generate a non-redundant database giving viral operational taxonomic units (vOTUs) which would represent the largest of many UViGs at 95% identity. BWA/SAMtools were then used for read-mapping, followed by analysing mapped viral reads and visualising the outcome of PCR and PCR-free datasets in relative abundance and alpha and beta diversity. In parallel, the qualities of both pooled non-redundant viral contigs from PCR and PCR-free datasets were assessed using CheckV, respectively. Moreover, DemoVir was used to assign taxonomy to these viral contigs, followed by performing cluster analysis to compare vOTU similarity between both datasets using vConTACT v2.0. For unknown viral sequences which cannot be identified and annotated by DemoVir, vConTACT v2.0 was used to infer their taxonomy at the genus or family level. In addition, ViromeQC was used to evaluate our VLP isolation protocol, based on the extent of VLP enrichment.

An overview of the pipeline is shown in Figure 2 comprising steps to remove human DNA, clean and trim low-quality remaining reads, assemble contigs, identify candidate viral contigs (UViGs), conduct read mapping, and to determine alpha and beta diversity and assign taxonomy to vOTUs.

#### 3.3.1. Quality and Quantity of Raw Sequencing Output

**Figure 3:**
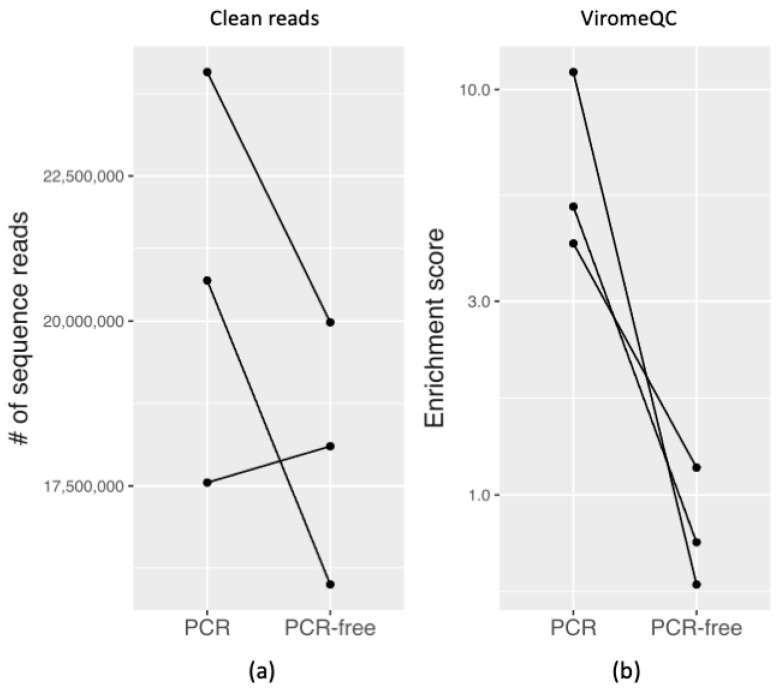
Quantity of cleaned reads and the measurement of VLP enrichment. The plots show the trends between the PCR and PCR-free groups. (**a**) The total cleaned sequence reads show that for samples 2 and 3 more reads were obtained from PCR compared to PCR-free samples, with insufficient evidence to detect an average effect on the number of reads (paired *t*-test *p*-value = 0.234). (**b**) From the ViromeQC analysis, the enrichment score was noticeably higher in PCR samples, on a log scale. However, the paired *t*-test on the log scale values were not statistically significant (*t*-test *p*-value = 0.05123). # represents “number”.

Using our optimised protocols for isolating faecal VLPs and VLP DNA (Figure 1), three DNA samples of high quality and quantity were aliquoted for library preparation and sequencing. For sequencing library preparation using PCR-based and PCR-free protocols, a total of six shotgun metagenomic DNA libraries (PCR_1-3 and PCR-free_1-3 datasets) were sequenced to generate a total of 54,202,065 cleaned reads from PCR-free libraries (18,067,355 ± 1,914,183.5, mean ± S.D; *n* = 3) and 62,693,258 cleaned reads from PCR libraries (20,897,753 ± 3,471,445.7, mean ± S.D; *n* = 3) (Table 1). The data generated had a >90% Q30 Phred score. In samples 2 and 3 the PCR library datasets produced more reads than the PCR-free library, but in sample 1 the PCR-free library produced more. The plot of the number of clean reads can be seen in Figure 3a.

#### 3.3.2. Evaluating VLP Enrichment

To evaluate the extent of faecal VLP enrichment in the sequence libraries, the VLP-enrichment score of PCR-based virome datasets estimated by ViromeQC indicated virome-enriched samples, with 11.04 (PCR-1), 4.16 (PCR-2) and 5.13 times (PCR-3) compared to lower enrichment scores of non-amplified virome datasets ranging from 0.6 to 1.17. When compared to a baseline score of 1 for a metagenome (Table S2) this suggests that the PCR-free samples were not considered to be enriched by the ViromeQC algorithm.

#### 3.3.3. Quality and Quantity of Assembled Contigs

Cleaned reads of each dataset were individually assembled into contigs yielding 280,041 contigs from PCR-free datasets and 208,529 contigs from PCR datasets. Across both library datasets, sample 1 produced 173,739 contigs, sample 2 produced 234,574 contigs and sample 3 produced 80,257 contigs. Of these, PCR-free datasets yielded 50,707 contigs of length ≥ 1 kbp across all samples, while PCR datasets produced 36,848 contigs of length ≥ 1 kbp. Combining PCR and PCR-free, sample 1 generated 32,420 contigs of length ≥ 1 kbp, sample 2 generated 44,366 contigs of length ≥ 1 kbp and sample 3 generated 10,769 contigs of length ≥ 1 kbp. In addition, in PCR-free datasets the mean of N50 per sample was 2313.3 ± 285.9 bp (mean ± S.D., *n* = 3), while in PCR datasets, the mean of N50 per sample was 3858.3 ± 2774.7 bp (mean ± S.D., *n* = 3) (Table 1).

#### 3.3.4. Identifying Putative Viruses

VirSorter and VirFinder were used to predict and identify putative non-cultivated viral genomes (UViGs; including provirus genomes) as described in the Materials and Methods section. In total 701 putative UViG contigs were identified in PCR datasets and 694 were identified in PCR-free datasets by VirSorter. Of these, 244 putative UViGs were present in PCR-1 dataset, 269 putative UViGs were found in PCR-2 dataset and 188 putative UViGs in PCR-3 dataset. In addition, 297 putative UViGs were detected in PCR-free-1 dataset, 221 putative UViGs were in PCR-free-2 dataset and 176 putative UViGs were in PCR-free-3 dataset. In total 27,832 putative UViG contigs in the PCR datasets and 34,418 putative UViG contigs in PCR-free datasets were predicted by VirFinder. Of these, 8609 putative UViGs were detected from PCR-1 dataset, 15,228 putative UViGs were found in PCR-2 dataset and 3995 putative UViGs were seen in PCR-3 dataset. In addition, 12,392 putative UViGs were found in PCR-free-1 dataset, 16,494 putative UViGs were in PCR-free-2 dataset and 5532 putative UViGs were in PCR-free-3 dataset (Table S3). Moreover, based on the parameters used, VirSorter-positive UViGs accounted for between 0.17% and 0.59% of the total assembled contigs, while the VirFinder-positive UViGs accounted for between 11.48% and 14.75% in total assembled contigs (Table S3).

UViGs were pooled and made non-redundant with 95% sequence identity over 90% of the contig length, resulting in clusters that corresponded to bacteriophage species, referred to as viral operational taxonomic units (vOTUs). Each vOTU is represented by the longest representative UViG. These vOTUs were used in downstream analysis. We found that the number of vOTUs with a length of > 1 kbp was 17,898 and 19,591 in PCR datasets and PCR-free datasets, respectively (Table S4). CheckV analysis was performed on these vOTUs > 1 kbp to assess their genome completeness and quality (Table S5).

#### 3.3.5. Read Mapping

Cleaned reads from each dataset were mapped against the reference vOTUs. Overall, the proportion of cleaned viral reads represented by non-redundant vOTUs varied between 72% and 91% (Table S6). PCR-3 and PCR-free-3 datasets yielded the maximum absolute numbers of mapped viral reads, compared with other datasets of samples 1 and 2 while the PCR-1 and PCR-free-1 datasets had higher mapping rates against the vOTUs, reaching up to 90%. PCR-free-2 datasets yielded the fewest absolute number of mapped viral reads with both PCR-2 and PCR-free-2 datasets having minimum mapping rates to the vOTUs, when compared with other datasets.

### 3.4. Comparing Virome-Derived PCR and PCR-Free Datasets: Relative Abundance Analysis

**Figure 4:**
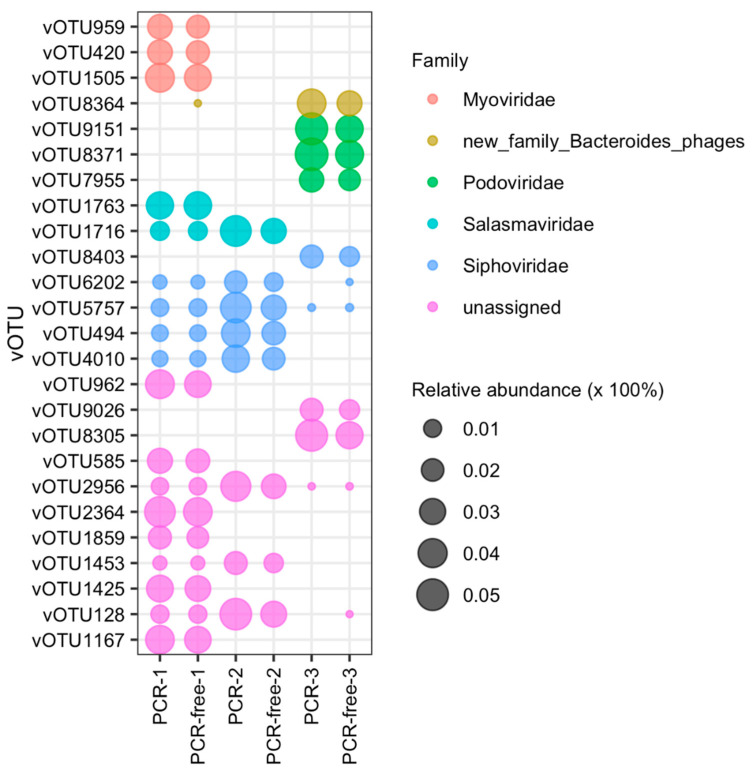
Relative abundance of the top 25 vOTUs from the PCR and PCR-free datasets. Bubble plot showing the relative abundance of the top 25 vOTUs across the datasets with assigned and unassigned taxonomies. Those vOTUs that could not be classified and assigned to appropriate taxonomies by either DemoVir or vConTACT 2.0 were labelled as “unassigned”. The size of bubble represents the relative abundance expressed as percentage (e.g., 0.02 means 2%) and each assigned or unassigned family group was separated in colour.

To investigate if PCR amplification impacted VLP-enriched intestinal/faecal viromes relative abundances were compared. Figure 4 displays the top 25 most abundant vOTUs across the datasets as inferred from read mapping results (see methods), indicating that the intestinal/faecal virome profiles were considerably different and unique in each individual donor. Overall, the virome composition was comparable between PCR and PCR-free methods from the same donor but varied considerably between individual donors. However, when comparing PCR and PCR-free methods we observed fine-scale differences between the two. In sample 1, a genome (vOTU8364) assigned to a potential new family of *Bacteroides*-associated phages was only seen in the PCR-free-1 and not in the PCR-1 vOTUs. Differences in the abundance of other vOTUs were also found in sample 1 when comparing the PCR to PCR-free dataset. In sample 2, no additional vOTUs were seen in either PCR or PCR-free datasets amongst the top 25 vOTUs. In sample 3, vOTU6202 assigned to the family *Siphoviridae* and vOTU128 assigned to a new family VC_442 were only seen in PCR-free-3 dataset and not in PCR-3. Overall, from the top 25 vOTUs, the most abundant vOTUs across the samples were assigned to the family *Siphoviridae*, followed by *Salasmaviridae* seen in both sample 1 and 2, and *Podoviridae* seen in the sample 3. Interestingly, *Myoviridae* was the least abundant family and was only seen in sample 1. Of note, the families *Myoviridae*, *Podoviridae* and *Siphoviridae*, which are scheduled to be abolished, are not genomically cohesive and can group phages that share no orthologous genes, whereas the family *Salasmaviridae* is a family of small podoviruses recently established [47]. The PCR samples in general had a higher relative abundance of the top 25 vOTUs.

#### 3.4.1. Alpha Diversity Analysis

**Figure 5:**
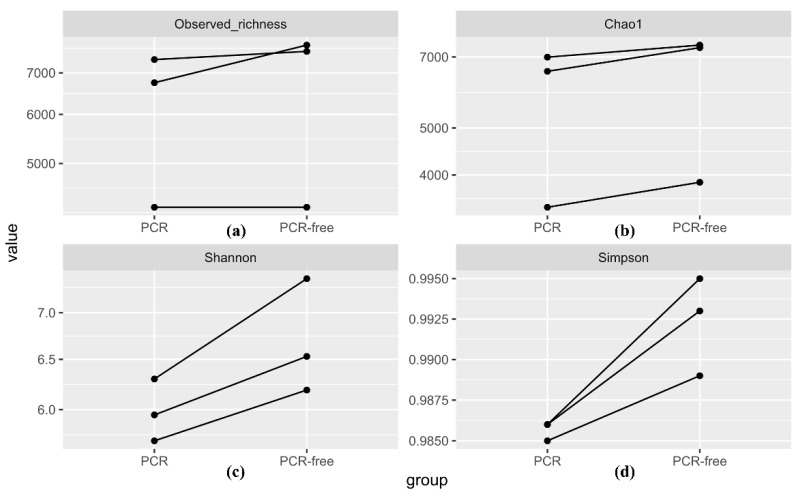
Estimations of the alpha diversity indices. (**a**) The number of vOTUs directly observed from a rarefied count matrix (*p*-value = 0.310). (**b**) Estimation of richness using Chao1 index for six virome-derived library datasets (*p*-value = 0.045). (**c**) Estimation of Shannon index for the virome datasets (*p*-value = 0.063). (**d**) Estimation of Simpson index for the virome datasets (*p*-value = 0.044). The *p*-values were obtained using a paired *t*-test analysis.

To compare intra-subject differences within each donor’s sample, “observed richness”, “Chao1”, “Shannon” and “Simpson” indices were used to measure the species richness and alpha diversity in each dataset based on all rarefied count matrices of vOTUs (Figure 5).

For observed richness (Figure 5a), the numbers of vOTUs in PCR-free datasets (7760 in PCR-free-1, 7581 in PCR-free-2 and 4250 in PCR-free-3) were higher than those in PCR datasets, albeit this difference was not statistically significant (6749 in PCR-1, 7354 in PCR-2 and 3795 in PCR-3) (*p*-value = 0.310).

Using the Chao1 estimator (Figure 5b), the richness estimates in sample 1 were 6540 ± 67 in PCR-1 vs. 7315 ± 60 in PCR-free-1 (mean ± S.E.M.), while the Chao1 estimates of PCR-free-2 and PCR-free-3 datasets were higher than those of PCR-2 and PCR-3 datasets (*p*-value = 0.045).

For Shannon index estimations of both richness and evenness (Figure 5c), the estimates of PCR-free datasets (6.19 in PCR-free-1, 7.39 in PCR-free-2 and 6.53 in PCR-free-3) were higher than those of PCR datasets, although this difference was not statistically significant (5.71 in PCR-1, 6.30 in PCR-2 and 5.95 in PCR-3) (*p*-value = 0.063).

Finally, from the Simpson index scores (Figure 5d), the evenness estimates of PCR-free datasets (0.989 in PCR-free-1, 0.995 in PCR-free-2 and 0.993 in PCR-free-3) were higher than those of PCR datasets (0.985 in PCR-1, 0.986 in PCR-2 and 0.986 in PCR-3) (*p*-value = 0.044).

Collectively, there is some evidence that the alpha diversity and species richness estimates of the intestinal/faecal viromes were higher among PCR-free datasets than PCR datasets within each donor’s sample. The *p*-value test shows this to be generally true for two of the indices used; this could be a limitation of the sample size used.

#### 3.4.2. Beta Diversity Analysis

**Figure 6:**
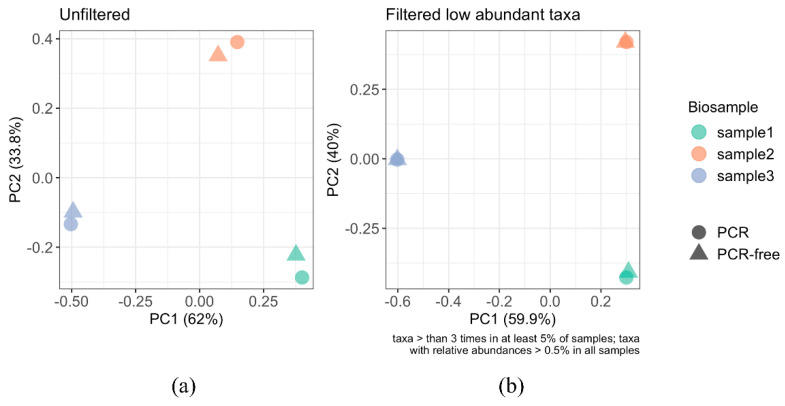
PCoA analysis of the faecal (intestinal) viromes from PCR and PCR-free datasets. The PCoA plots were based on Bray–Curtis distances and were used to interpret the intestinal/faecal virome-derived datasets. (**a**) PCoA of the unfiltered data and (**b**) PCoA plot after removing <0.5% abundant taxa.

To evaluate the impact of amplification on the whole viral community, we compared beta diversity by computing the distance matrices of Bray–Curtis dissimilarities among all datasets (Figure 6). The unfiltered PCoA (Figure 6a) revealed that the dissimilarity distance between donors was greater than that between the PCR and PCR-free for each donor. This indicates that inter-donor differences in the virome have a greater effect than PCR or PCR-free processing. The subtle dissimilarity between PCR and PCR-free processing observed in the unfiltered PCoA were not apparent when filtering out low abundant vOTUs and rare taxa (relative abundance below 0.5%) (Figure 6b).

### 3.5. Viral Clustering and Taxonomy: Sequence Similarity Networks

**Figure 7:**
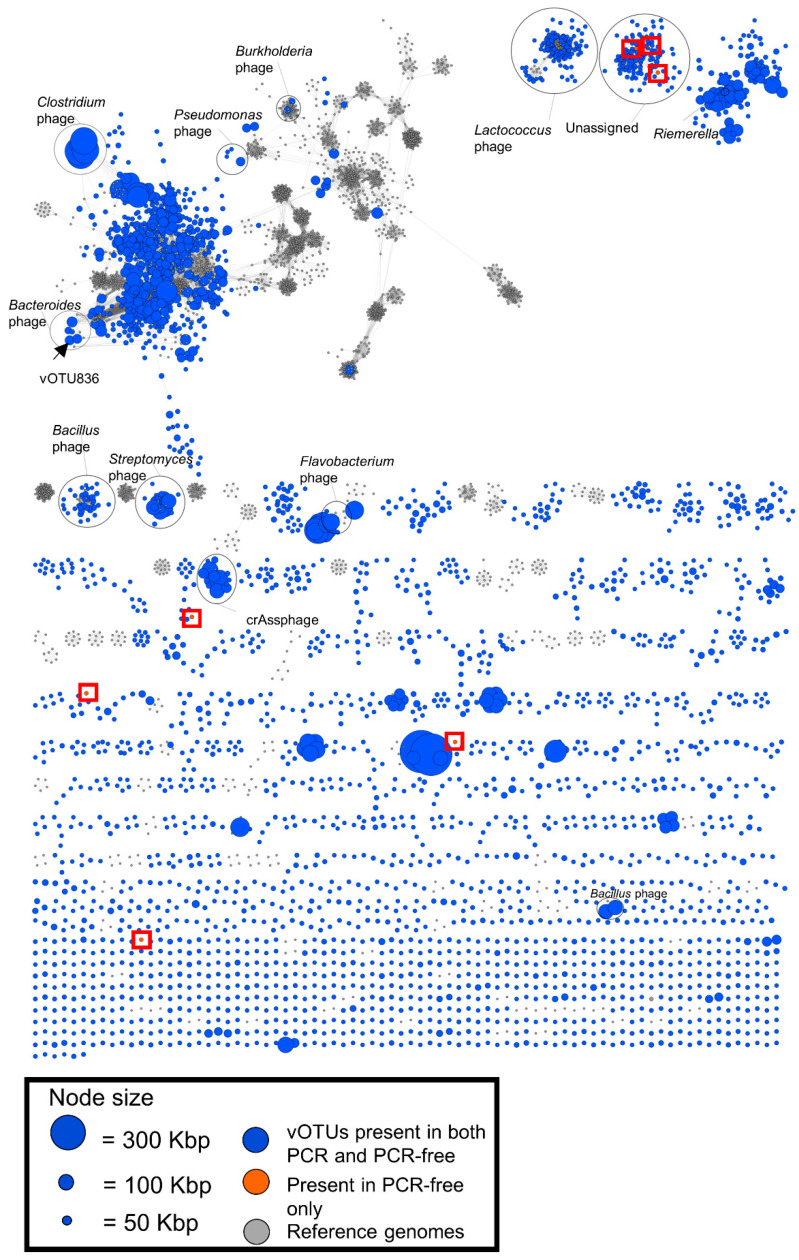
Viral cluster network displaying the non-redundant viral sequence similarity for both the PCR and PCR-free datasets. vOTUs are grouped into a viral cluster with shared sequence similarity based on amino acid homology against reference genomes (grey circles). The nodes (blue circles) represent vOTUs and edges (grey lines) represent the weighted similarity between the vOTUs. The red boxed orange nodes, were only present in PCR-free datasets.

To further investigate the similarity of vOTU with a length > 1 kbp between PCR and PCR-free datasets and to infer taxonomy, a genome-based network analysis was carried out using vConTACT 2.0 [39,40]. Each vOTU was clustered with reference phage genomes (ProkayoticViralRefSeq94) into viral clusters (VCs) based on shared gene content. The genomes (Figure 7: blue for vOTU, grey for reference genomes) are displayed as nodes connected by edges if they share a significant proportion of their predicted protein content. 1358 VCs clusters had a direct link to a reference genome but were distant to other phages. The complex clustering in the top left corner of the map were linked to reference phage genomes of *Streptococcus*, *Lactobacillus*, *Bacillus*, *Staphylococcus*, *Listeria*, *Enterococcus* and *Brevibacillus*. Based on the abundance data of the non-redundant vOTU catalogue > 1 kbp, 75 contigs were only present in either PCR or PCR-free datasets (Table S7). Moreover, the network analysis identified seven vOTUs (Figure 7, orange nodes) that were only present in the PCR-free datasets. Of these, three were part of an unassigned cluster in the top right of the network (VCs 520 and 523), three had connections to VCs 556,719 and 640, and one had a connection to VC 1344. There were no reference phages associated with these seven vOTUs. The remaining 68 vOTUs showed no similarity to other vOTUs or the reference genomes and were therefore not included in the network.

## 4. Discussion

To address how amplification bias during sequence library preparation impacts the human faecal virome we optimised a VLP/DNA isolation protocol and selected suitable bioinformatics tools to compare PCR-based LASL (linker amplified shotgun library) to NASL (non-amplified shotgun library) methods for Illumina sequencing library preparation. Our analysis shows that PCR amplification introduces biases particularly with respect to measures of viral relative abundance and alpha diversity. The biases were more apparent when examining less abundant genomes. Thus, differences in methods are particularly important when investigating the “rare” members of the virome and should be considered when diversity metrics are calculated.

Our VLP isolation protocol was optimised using samples spiked with a reference phage to assess the efficiency of recovery. To prevent VLP damage and maximise recovery, faecal samples were homogenised by hand-mixing and vortexing followed by using high-speed centrifugation to remove debris and bacteria, accepting that giant viruses and/or viral aggregates may also be lost. A similar compromise was made regarding filtration using a combination of 0.8 μm and 0.45 μm filters which, while effective at removing bacterial contaminants, negatively impact virus recovery (30–40% recovery of reference phage). The majority of VLPs recovered comprised intact VLPs and detached viral capsids, which was supported by TEM, meaning that while our VLP isolation protocol was efficient and well-suited to recovering viral capsids for downstream molecular-based virome analysis it is less well-suited to recovering fully intact, infective viruses (phages).

Overall, although the number of reads produced from PCR datasets was higher than those produced from PCR-free datasets, a similar number of cleaned reads was expected from all libraries as equimolar amounts were loaded for sequencing to generate similar target sequence yields. However, limited input of viral DNA is often seen in virome studies, particularly for those using clinical samples. Low amounts of viral DNA (input of a few nanograms or less) is likely to cause bias when using a PCR-based LASL protocol [20,48,49]. Using the LASL method may however be unavoidable due to sample limitations. Omitting PCR amplification steps requires relatively large amounts of input viral DNA for library preparation and Illumina sequencing, which makes this approach impractical for many types of clinical samples [50]. In addition, biases are likely to be introduced from elsewhere during sequencing making it difficult to subsequently remove biases from Illumina sequence datasets [50]. Hence, although omitting PCR steps during library preparation is ideal to reduce bias, it is often ignored.

ViromeQC working on raw paired-end sequencing reads followed by sorting high-quality reads was used to calculate the abundance of microbial markers and generate an enrichment score for each virome dataset. ViromeQC compared the abundance of these microbial markers to a default baseline score of one which was computed on 2000 metagenomes [29]. Although a custom value can be provided, we used this default base score. The ViromeQC scores in PCR datasets reached 11-fold in sample 1. In contrast, the enrichment scores in PCR-free datasets were 0.5- to 1.2-fold compared to non-enriched reference metagenomes. An important consideration in using ViromeQC is that it is trained for enriched viral DNA samples generated by amplification-based library preparation using a standard protocol. ViromeQC gave lower scores to the PCR-free sample as they had more LSU rRNA marker present. Despite the enrichment score of the non-amplified samples being relatively low, UViGs were detectable in our analysis, as reported by others [51]. In addition, even though the datasets displayed in Figure 3b suggest a difference between PCR and PCR-free treatments, a paired *t*-test analysis showed that these differences were not significant. Investigating the disparity with ViromeQC further, it was determined that some reads in the PCR-free datasets mapped to SILVA LSU rRNA (Table S2) and mapped predominantly but not exclusively to *Weissella viridescens* (accession: CYXF01000014). These reads were likely due to the PCR-free method of library construction and the sequence depth used in this study. We infer that the presence of bacterial genomes in the PCR-free dataset is not only due to contamination, but also bacteriophages that harbour and spread bacterial genes through transduction [52].

Analysis of the relative abundance of the 25 most abundant vOTUs reveals that PCR and PCR-free library preparation are similar. Overall, the majority of the top 25 vOTUs were seen in both PCR and PCR-free derived datasets. However, viruses potentially assigned to a potentially new family of *Bacteroides* phages were only seen in the PCR-free-1 and not in the PCR-1 vOTUs (vOTU8364), whereas siphovirus vOTU6202 and a new unassigned family of vOTU128 were not seen in PCR-3 dataset. Of these differences, the relative abundance of vOTUs in all PCR datasets was higher than in PCR-free datasets (Figure 4 and Table S7), indicating that PCR amplification by the LASL method influences viral representation causing over- or under-estimation for a number of viral taxa, as seen in a recent virome study evaluating SISPA (sequence-independent, single-primer amplification) and MDA-based random amplification of the human saliva virome [20]. However, we could not discern any systematic over- or underrepresentation at the vOTU, VC or viral families. Moreover, the top nine vOTUs seen in each dataset could not be given a taxonomy assignment using either DemoVir or vConTACT 2.0, suggesting that these are likely to be novel taxa. In future, and given the advances in phage taxonomy, these vOTUs could be assigned to new, as-of-yet undefined genome-based phage families [47]. At this time, a tool like DemoVir can be used to identify a contig as a tailed phage (class *Caudoviricetes*) but will not be able to accurately predict the taxonomic assignments below as these consist of a mixture of genome- and morphology-based families at the time of analysis [47]. Gene-sharing network tools such as vConTACT2 can in the meantime be used to reveal the relationships between vOTUs and reference databases, without providing an official taxonomic assignment. Metagenomic virus classification could be improved by the incorporation of several published non-redundant viral reference databases, such as NCBI Viral RefSeq [53], the human gut virome database (GVD) [5], the integrated microbiome genome/virus system (IMG/VR) [54,55], the gut phage database [3] and/or a new reference viral database (RVDB) [56] to shed further light on “viral dark matter”. Consistent with our TEM analysis, the relative abundance analysis indicated that siphovirus morphology was the most abundant virus morphotype seen in all individual samples (Figure 4 and Figure S1). Interestingly, although in the relative abundance analysis (top 25 vOTUs) myoviruses were only seen in donor 1, they could also be observed in donors 2 and 3 by TEM, indicating high individuality in the human faecal viromes, as noted by others [6,57].

The alpha diversity of the PCR-free datasets was higher than those in PCR datasets for the Simpson index and Chao1 estimator (Figure 5). The Shannon index although not significant (*p* = 0.063) showed a similar trend. This suggests that PCR amplification affects the distributions of the human faecal viromes and is likely to lower their richness and alpha diversity. In each of the three samples, although our rarefied alpha-diversity measures were higher among PCR-free datasets compared to PCR datasets, statistical tests only provided some evidence that this finding was generally true although our study was not designed to test this hypothesis. There was a high degree of inter-sample variation in both the alpha and beta diversity evident from the PCoA plot using the Bray–Curtis dissimilarity matrix on unfiltered (Figure 6a) and filtered (Figure 6b) datasets set to >0.5% relative abundance. Both ordination plots showed that the locations between sample clusters were farther apart than those between PCR and PCR-free datasets in each sample, implying that the major difference in inter-subject beta diversity was driven by high specificity of the intestinal/faecal viromes in each subject [6,57]. Furthermore, when filtering to only include relative abundance of >0.5%, the difference between PCR and PCR-free was negligible. This supports the hypothesis that PCR-free samples have a higher species richness as a result of resolving low abundance taxa, resolvable at read depths of ~4.9 Gbp to 7.3 Gbp as in this study.

Finally, viral cluster (VC) and taxonomy network analysis was performed to group vOTUs into diverse VCs based on genome similarity and to investigate the strength of relationships between VCs based on their amino acid homology. In an initial cluster analysis, we found that although all vOTUs were grouped into diverse VCs there was no significant difference between the PCR and PCR-free datasets, with both having shared vOTU sequences (Figure 7). This is presumably explained by the fact that both datasets were derived from the same DNA samples and some vOTUs may be conserved across the samples, such as crAssphage, which tend to be conserved over time in the human population, particularly in healthy individuals [6]. CrAss-like vOTUs are seen in Figure 7 but are absent from Figure 4, as they were not part of the top 25 most abundant in our cohort. Some of the crass-like vOTUs present in Figure 7 appear when looking at the top 75 most abundant phages. Similarly, high virome stability has also been observed in a single healthy adult and in a twin cohort study [58,59]. However, as we saw a higher species diversity in the PCR-free samples and the viral network showed seven nodes only present in the PCR-free cohort, considerations should be made when trying to resolve the taxa present in the low-abundance viromes. In addition, many small or singleton VCs could be seen in both datasets displaying either no or few connections to each other. Most of the singleton VCs resulted from small (<1 kbp) viral contigs, suggesting that sorting contig length to >2 kbp or >5 kbp is required to reduce network complexity in future studies. Selecting UViGs >10 kbp has been considered in a recent virome study [5].

## 5. Conclusions

Our findings demonstrate that most variations between the PCR and PCR-free library preparation are interpreted as inter-individual differences, reflecting a unique composition of the faecal virome between individuals. However, there is evidence that use of PCR amplification during library preparation introduces biases that may have an impact on viral relative abundance and alpha diversity, particularly on detecting low-abundance viral taxa, thereby possibly leading to misinterpretation on the “rare” members of the virome. We therefore recommend, whenever possible, to use amplification-free protocols in virome studies to minimise biases.

## Figures and Tables

**Table 1:** Sequence read quality and quantity of PCR and PCR-free datasets.

Donor #	PCR Library			PCR-Free Library		
	1	2	3	1	2	3
**# Raw reads**	17,618,631	20,715,201	24,640,272	18,647,093	16,540,559	20,897,792
**# Cleaned reads**	17,547,247	20,667,351	24,478,660	18,072,149	16,150,779	19,979,137
**Q30 (%)**	90.29	92.47	92.22	93.62	90.91	90.72
**% GC**	36.05	46.89	38.48	36.79	46.71	39.42
**# Contigs**	73,246	103,220	32,063	100,493	131,354	48,194
**# Contigs ≥ 1 kbp**	14,469	17,952	4427	17,951	26,414	6342
**N50 (bp)**	2011	2515	7049	2019	2331	2590

# represents “number” or “number of”.

## Data Availability

The SRA have been deposited to ENA under accession … and the nr-contigs have been deposited under the study accession PRJEB47625.

## Supplementary Materials

The following are available online at https://www.mdpi.com/article/10.3390/v13102093/s1, Figure S1: Transmission electron micrographs of VLPs after dual 0.8 and 0.45 µm filtration, Table S1: Recovery of (spiked) phage and VLP DNA from processed human faeces after dual filtration, Table S2: Evaluation of VLP enrichment using ViromeQC, Table S3: Putative UViGs detected by VirSorter and VirFinder, Table S4: Quantity of non-redundant vOTUs > 1 kbp, Table S5: CheckV output on non-redundant vOTUs > 1 kbp, Table S6: Statistics of mapped and unmapped reads from PCR and PCR-free library datasets to vOTUs, Table S7: Abundance counts of viral contigs, vOTU’s assignment of Top 25 UViGs in each sample and the taxonomic assignment of the top 25 most abundant vOTUs and Method S1: Epifluorescence Microscopy (EFM)-based Phage Spiking and Recovery [60].
