# Supplementary material for: Comparison of PCR versus PCR-Free DNA Library Preparation for Characterising the Human Faecal Virome
Source: Viruses. 2021 Oct 18;13(10):2093. doi: 10.3390/v13102093 (PMC8537689; doi:10.3390/v13102093)
Supplement: Supplementary file 1 [file viruses-13-02093-s001.zip › supplementary material/FS1.pdf]

Figure S1: Transmission electron micrographs of VLPs after dual 0.8- and 0.45-µm filtration.

Red arrows indicate intact virions and virus-like structures. (A) siphovirus (capsid: ~85.7 nm in diameter; tail: ~285.7 nm in length), (B) siphovirus (capsid: ~166.7 nm in diameter; tail: ~566.7 nm in length), (C) siphovirus (capsid: ~80 nm in diameter; tail: ~180 nm in length), (D) myovirus associated with membranous material (capsid: ~100 nm in diameter; tail: ~100 nm in length), (E) myovirus (capsid: ~100 nm in diameter; tail: ~200 nm in length). Scale bar: 100-200 nm.

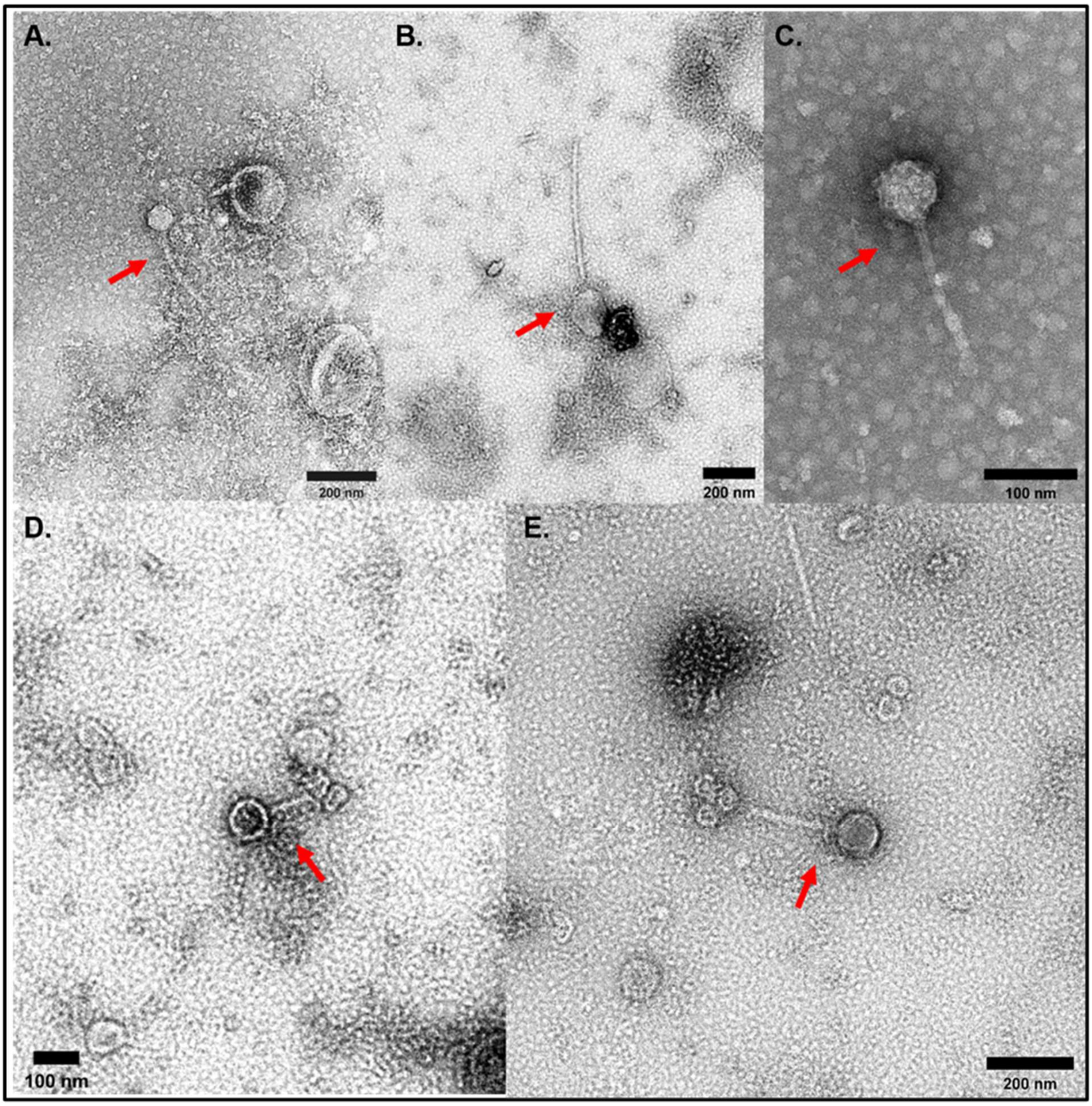

Figure S1: *Transmission electron micrographs of VLPs after dual 0.8- and 0.45-µm filtration.*
Red arrows indicate intact virions and virus-like structures. (A) siphovirus (capsid: ~85.7 nm in diameter; tail: ~285.7 nm in length), (B) siphovirus (capsid: ~166.7 nm in diameter; tail: ~566.7 nm in

length), (C) siphovirus (capsid: ~80 nm in diameter; tail: ~180 nm in length), (D) myovirus associated with membranous material (capsid: ~100 nm in diameter; tail: ~100 nm in length), (E) myovirus (capsid: ~100 nm in diameter; tail: ~200 nm in length). Scale bar: 100-200 nm.
