# Supplementary material for: Comparison of PCR versus PCR-Free DNA Library Preparation for Characterising the Human Faecal Virome
Source: Viruses. 2021 Oct 18;13(10):2093. doi: 10.3390/v13102093 (PMC8537689; doi:10.3390/v13102093)
Supplement: Supplementary file 1 [file viruses-13-02093-s001.zip › supplementary material/MS1.pdf]

Method S1: *Epifluorescence Microscopy (EFM)-based Phage Spiking and Recovery*

Phage stock (900 µl) was incubated with 100 µl of 0.25 % (v/v) SYBR Gold (Thermo Fisher Scientific, UK) prior to use in spiking assays. 2 x 10 g of fresh faeces (~20 g in total) from sample 1 were homogenised in 25 ml of TBT buffer using vortexing. Faecal homogenates were pooled (~50 ml) and then spiked with 1 ml of SYBR Gold (final concentration 0.025 %, v/v) labelled ΦB124-14 phage, followed by centrifugation, dual filtration and PEG precipitation as described above. Phage-containing precipitates were resuspended in 2 ml of TBT buffer with 20 µl of the PEG-VLP suspension diluted 1:50 (v/v) in nuclease-free water prior to immobilization on filter membranes. Briefly, an Omnipore 0.45 µm, 13-mm PTFE backing filter (Millipore/Sigma-Aldrich Ltd., UK) was placed on top of a Swinnex filter holder with a silicone gasket (Millipore/Sigma-Aldrich Ltd., United Kingdom). The backing filter was rinsed with nuclease-free water followed by placing a 0.02 µm white WhatmanTM Anodisc 13-mm filter membrane (Sigma-Aldrich Ltd., UK) placed on top of the backing filter. The Anodisc filter membrane was rinsed by nuclease-free water using a low-vacuum pressure (2-4 psi or ~20 kPa; Millivac-Maxi vacuum pump, Millipore/Sigma-Aldrich Ltd., UK), as described previously [11,60]. The phage-spiked sample (20 µl) was then fixed on the Anodisc membrane using low vacuum pressure until all liquid had passed through the membrane. The membrane was washed with 1 ml of nuclease-free water to remove excess dye. The filter membrane was then transferred to a Whatman® filter paper disc (Sigma-Aldrich, United Kingdom) and left to dry for 60 s. Prior to adding a coverslip, a small drop of Fluoromount-G® antifade mounting reagent (SouthernBiotech, US) was spotted on a microscope slide and the dried filter membrane was then placed onto the mounted droplet. The slide was left at 20 °C in the dark for 16 h and slides were subsequently imaged using a Zeiss Axio Imager M2 widefield epifluorescence microscope with the Alexa Fluor 488 channel and the 100 high-resolution oil objective lens. For each slide 20 digital images were captured and SYBR Gold-labelled viral particles were viewed and enumerated using ImageJ software. To enumerate the spiked viral particles, the average number of VLPs per field was multiplied by sample dilution factor and microscope conversion factor (i.e. area of 13-mm Anodisc filter / area of field of view), and then divided by the sample volume, as described previously [60].
