# Supplementary material for: Comparison of PCR versus PCR-Free DNA Library Preparation for Characterising the Human Faecal Virome
Source: Viruses. 2021 Oct 18;13(10):2093. doi: 10.3390/v13102093 (PMC8537689; doi:10.3390/v13102093)
Supplement: Supplementary file 1 [file viruses-13-02093-s001.zip › supplementary material/TS1.pdf]

**Table S1.** *Recovery of (spiked) phage and VLP DNA from processed human faeces after dual filtration*

| Donor # | Sample type | Sample weight (g) | Faecal aliquots # | PFU recovery (%)* | % Overall recovery (mean ± SD, n = 3) | DNA (ng/µl) | Total DNA (µg) | $OD_{260/280}$ | DNA yield (µg/g faeces) |
|---|---|---|---|---|---|---|---|---|---|
| 1 | Fresh | 47.0 | 12 | 34.5 | | 53.7 | 4.46 | 1.8 | 0.095 |
| 2 | Fresh | 37.5 | 10 | 32.6 | 35.8 ± 3.96 | 35.0 | 2.52 | 1.8 | 0.067 |
| 3 | Frozen | 37.0 | 10 | 40.2 | | 46.4 | 3.20 | 1.8 | 0.086 |

*As determined by dividing PFU of pure phage stock (φB124-14) into PFU after PEG precipitation then multiplying by 100 %. All PFU recovery was replicated three times for each sample.
