# Supplementary material for: Comparison of PCR versus PCR-Free DNA Library Preparation for Characterising the Human Faecal Virome
Source: Viruses. 2021 Oct 18;13(10):2093. doi: 10.3390/v13102093 (PMC8537689; doi:10.3390/v13102093)
Supplement: Supplementary file 1 [file viruses-13-02093-s001.zip › supplementary material/TS2.pdf]

Table S2. *Evaluation of VLP enrichment using ViromeQC*

Sample	Raw reads	Reads_HQ	SSU rRNA alignment rate	LSU rRNA alignment rate	Bacterial markers alignment rate	Total enrichment score
PCR-free-1	37,294,186	37,140,565	0.033	0.884	0.088	0.600
PCR-free-2	33,081,118	32,889,577	0.136	0.454	0.257	1.168
PCR-free-3	41,795,584	41,435,311	0.098	0.695	0.164	0.763
PCR-1	35,237,262	34,998,689	0.022	0.041	0.063	11.041
PCR-2	41,430,402	41,300,288	0.059	0.108	0.166	4.162
PCR-3	49,280,544	49,006,610	0.048	0.088	0.096	5.131

HQ: high-quality; SSU-rRNA: small subunit rRNA gene; LSU-rRNA: large subunit rRNA gene
