# Supplementary material for: Comparison of PCR versus PCR-Free DNA Library Preparation for Characterising the Human Faecal Virome
Source: Viruses. 2021 Oct 18;13(10):2093. doi: 10.3390/v13102093 (PMC8537689; doi:10.3390/v13102093)
Supplement: Supplementary file 1 [file viruses-13-02093-s001.zip › supplementary material/TS3.pdf]

Table S3. *Putative UViGs detected by VirSorter and VirFinder*

Dataset	# Total assembled contigs	# VirSorter-positive	% VirSorter-positive*	# VirFinder-positive	% VirFinder-positive*
PCR-1	73,246	244	0.33	8,609	11.75
PCR-free-1	100,493	297	0.30	12,392	12.33
PCR-2	103,220	269	0.26	15,228	14.75
PCR-free-2	131,354	221	0.17	16,494	12.56
PCR-3	32,063	188	0.59	3,995	12.46
PCR-free-3	48,194	176	0.37	5,532	11.48

*As determined by dividing the number of each assembly into the number of VirSorter- or VirFinder-positive then multiplying by 100 %
