# Supplementary material for: Comparison of PCR versus PCR-Free DNA Library Preparation for Characterising the Human Faecal Virome
Source: Viruses. 2021 Oct 18;13(10):2093. doi: 10.3390/v13102093 (PMC8537689; doi:10.3390/v13102093)
Supplement: Supplementary file 1 [file viruses-13-02093-s001.zip › supplementary material/TS4.pdf]

Table S4. *Quantity of non-redundant vOTUs > 1 kbp*

	**PCR-1**	**PCR-free-1**	**PCR-2**	**PCR-free-2**	**PCR-3**	**PCR-free-3**
# vOTUs (> 1 kbp)	6,749	7,760	7,354	7,581	3,795	4,250
