# Supplementary material for: Comparison of PCR versus PCR-Free DNA Library Preparation for Characterising the Human Faecal Virome
Source: Viruses. 2021 Oct 18;13(10):2093. doi: 10.3390/v13102093 (PMC8537689; doi:10.3390/v13102093)
Supplement: Supplementary file 1 [file viruses-13-02093-s001.zip › supplementary material/TS6.pdf]

Table S6. *Statistics of mapped and unmapped reads from PCR and PCR-free library datasets to vOTUs*

	**PCR-1**	**PCR-free-1**	**PCR-2**	**PCR-free-2**	**PCR-3**	**PCR-free-3**
# of cleaned reads (paired-ended)	33,872,924	36,122,668	40,749,416	32,048,918	47,904,786	40,102,922
# of reads mapped to vOTUs	30,533,123	31,754,308	32,885,103	23,357,458	42,241,639	33,515,173
Mapping rate to vOTUs (%)	90.1 %	87.9 %	80.7 %	72.9 %	88.2 %	83.6 %
# of unmapped reads	3,339,801	4,368,360	7,864,313	8,691,460	5,663,147	6,587,749
Unmapping rate (%)	9.9 %	12.1 %	19.3 %	27.1 %	11.8 %	16.4 %
